# Sarcopenia and serum biomarkers of oxidative stress after a 6-month physical activity intervention in women with metastatic breast cancer: results from the ABLE feasibility trial

**DOI:** 10.1007/s10549-021-06238-z

**Published:** 2021-05-19

**Authors:** Lidia Delrieu, Agnès Martin, Marina Touillaud, Olivia Pérol, Magali Morelle, Olivia Febvey-Combes, Damien Freyssenet, Christine Friedenreich, Armelle Dufresne, Thomas Bachelot, Pierre-Etienne Heudel, Olivier Trédan, Hugo Crochet, Amine Bouhamama, Frank Pilleul, Vincent Pialoux, Béatrice Fervers

**Affiliations:** 1 Inter-University Laboratory of Human Movement Biology EA7424, University Claude Bernard Lyon 1, University of Lyon, 29 Boulevard du 11 Novembre 1918, 69622 Villeurbanne Cedex, France; 2 Department of Prevention Cancer Environment, Léon Bérard Cancer Centre, Lyon, France; 3 Inter-University Laboratory of Human Movement Biology EA7424, University of Lyon, University Jean Monnet Saint-Etienne, Saint-Etienne, France; 4 Inserm UA8, “Radiation: Defense, Health, Environment”, Léon Bérard Cancer Center, Lyon, France; 5 Department of Clinical Research and Innovation, Léon Bérard Cancer Centre, Lyon, France; 6 Department of Cancer Epidemiology and Prevention Research, CancerCare Alberta, Alberta Health Services, Calgary, AB Canada; 7 Departments of Oncology and Community Health Sciences, Cumming School of Medicine, University of Calgary, Calgary, AB Canada; 8 Data Factory Team, Léon Bérard Cancer Centre, Lyon, France; 9 Department of Medical Oncology, Léon Bérard Cancer Centre, Lyon, France; 10 Department of Radiology, Léon Bérard Cancer Centre, Lyon, France; 11 Institut Universitaire de France (IUF), Paris, France; 12 LabEx GR-Ex, Paris, France

**Keywords:** Metastatic breast cancer, Sarcopenia, Skeletal muscle index, Oxidative stress, Toxicity, Physical activity

## Abstract

**Purpose:**

Sarcopenia has been identified as an important prognostic factor for patients with cancer. This study aimed at exploring the potential associations between a 6-month physical activity intervention and muscle characteristics, sarcopenia, oxidative stress and toxicities in patients with metastatic breast cancer.

**Methods:**

Women newly diagnosed with metastatic breast cancer (*N* = 49) participated in an unsupervised, personalized, 6-month physical activity intervention with activity tracker. Computerized tomography images at the third lumbar vertebra were analysed at baseline, three months and six months to assess sarcopenia (muscle mass index < 40 cm^2^/m^2^) and muscle quality (poor if muscle attenuation < 37.8 Hounsfield Units). Oxidative markers included plasma antioxidant enzymes (catalase, glutathione peroxidase and superoxide dismutase activities), prooxidant enzymes (NADPH oxidase and myeloperoxidase activities) and oxidative stress damage markers (advanced oxidation protein products, malondialdehyde (MDA) and DNA oxidation.

**Results:**

At baseline 53% (mean age 55 years (SD 10.41)) were sarcopenic and 75% had poor muscle quality. Muscle cross sectional area, skeletal muscle radiodensity, lean body mass remained constant over the six months (*p* = 0.75, *p* = 0.07 and *p* = 0.75 respectively), but differed significantly between sarcopenic and non-sarcopenic patients at baseline and 6-months. Sarcopenic patients at baseline were more likely to have an increase of MDA (*p* = 0.02) at 6 months. Being sarcopenic during at least one moment during the 6-month study was associated with a higher risk of developing severe toxicities (grade > 2) (*p* = 0.02).

**Conclusions:**

This study suggests potential benefits of physical activity for maintenance of muscle mass. Sarcopenia can alter many parameters and disturb the pro and antioxidant balance.

**Supplementary Information:**

The online version contains supplementary material available at 10.1007/s10549-021-06238-z.

## Introduction

About 5% of breast cancers are metastatic at diagnosis and 20–30% of localized breast cancer become secondarily metastatic [1, 2]. The recent therapeutic advances have resulted in improvements in median survival which currently ranges from 2 to 3 years with a 5-year survival of ~ 25% in developed countries [1]. Metastatic breast cancer is considered incurable, and treatments are proposed to improve quality of life and overall survival. However, patients suffer from many detrimental symptoms, such as fatigue, pain, toxicities related to treatments and decrease in physical functioning related to treatment and metastasis [2, 3].

Sarcopenia is characterised by a decrease in skeletal muscle mass and in physical performance Sarcopenia is primarily associated with aging and is secondarily related with other chronic diseases. Sarcopenia is a key issue in cancer patients and has been identified as an important prognostic factor for patients with cancer [4–6]. Sarcopenia affects approximately 11% to 74% of cancer patients, depending on the site and severity of the tumours [7, 8]. A study of 166 women with metastatic breast cancer showed a prevalence of sarcopenia of 67% while another study of 40 patients showed a lower prevalence of 58% [9, 10]. In patients with advanced breast cancer, low muscle strength and sarcopenia have been associated with tumour progression and mortality [11–13]. In parallel, low muscle quality often reflects extensive lipid infiltration [14, 15]. One study found that low muscle radiodensity which is a measure of muscle quality indicative of adipose tissue deposition into muscle fibers and reduced function, increased risk of death [9]. Moreover, the literature has suggested associations between body composition and risk of treatment toxicities according to National Cancer Institute Common Toxicity Criteria for Adverse Events (NCI-CTCAE; Version 5.0) at early stages of cancer [16, 17], in advanced cancer [12, 13, 18–22] and specifically in breast cancer [7, 8, 22–26]. Several of these studies have demonstrated that the decrease in muscle mass was associated with an interruption of chemotherapy or a reduction in dose [21, 27, 28]. Because chemotherapy doses are determined from total body surface area, patients with lower muscle mass for a given total body surface area may receive an excessive dose of chemotherapy [12, 16, 19, 19, 29] and are more likely than their counterparts to develop toxicities [12, 19, 30–32]. The balance between reducing the risk of toxicity inherent to treatments and maintaining the efficacy of anticancer drugs raises the need to prevent sarcopenia by enhancing body composition.

Physical activity during and after cancer treatment for localized cancer has been shown to increase lean body mass [33, 34]. To our knowledge, only one randomized controlled study has analysed the effect of physical activity intervention on sarcopenia in patients with localized breast cancer during adjuvant chemotherapy [35]. This study has shown that a resistance training may be enable to offset the decrease in muscle strength and muscle mass (in particular through protein synthesis) and avoid sarcopenia [35, 36].

Preventing sarcopenia is a key factor in advanced cancer care, but reliable markers of sarcopenia have been difficult to identify [37, 38]. Oxidative stress defined as an imbalance between the production of reactive oxygen species (free radicals) and antioxidants defences in the body, has been suggested to be an early biomarker of sarcopenia [37, 38]. For example, lipid peroxidation markers have been positively correlated with sarcopenia [39]. A pro/antioxidant imbalance resulting from an excess of mitochondrial radical production associated with a decrease in antioxidant enzymes at the level of the muscle cell may be a major factor at the origin of the sarcopenia phenomenon [40]. In addition, sarcopenia can occur regardless of body mass index (BMI), in both lean and obese individuals, the latter constituting sarcopenic obesity [30].

The purpose of this ancillary study to the Advanced stage Breast cancer and Lifestyle Exercise (ABLE) Trial [41] was to (i) study the variation in muscle characteristics between baseline and the end of the 6-month physical activity intervention, (ii) explore the association between the variation between baseline and month six in muscle characteristics and physical activity level, physical fitness and quality of life, (iii) explore the association between sarcopenia at baseline and individuals characteristics, physical activity condition, oxidative stress at 6 months, and (iv) explore the association between sarcopenia during the study and toxicities during the study in patients with metastatic breast cancer. The ABLE Trial was not originally powered to detect changes in these outcomes and the present analysis was performed as a secondary objective to generate preliminary data on the association between physical activity, muscle characteristics, sarcopenia, oxidative stress and toxicities in metastatic breast cancer patients. We hypothesized that (i) the physical activity intervention would be positively associated with muscle mass and negatively with sarcopenia, (ii) sarcopenic participants would have higher risk of toxicity during time and a disturbed pro-oxidant/antioxidant balance at 6 months as compared to nonsarcopenic patients.

## Methods

### Study design

The ABLE trial that was registered on http://www.clinicaltrials.gov (clinicaltrials.gov identifier: NCT03148886). The study protocol has been described in detail elsewhere [41]. Briefly, ABLE is a single-arm intervention trial conducted at the Léon Bérard Comprehensive Cancer Centre (Lyon, France) in which patients newly diagnosed with metastatic breast cancer were followed for their physical activity, physical function, quality of life, fatigue and tumour progression during a 6-month unsupervised intervention in physical activity.

The study protocol was approved by the local research ethics committee (Comité de Protection des Personnes Sud-Est IV) and the study database was reported to the National Commission for Data Protection and Liberties (CNIL) (reference number: 1994192).

### Recruitment of participants

Patients were female, aged 18 to 78 years; newly diagnosed with a de novo or secondary metastatic breast cancer histologically confirmed within the last 3 months; treated with any combination of chemotherapy, radiotherapy, hormonal therapy and targeted therapy; having an Eastern Cooperative Oncology Group (ECOG) Performance status < 2; having a medical clearance of no contraindications to physical activity; able to speak and understand French; able to complete questionnaires and follow instructions in French; and having a valid health insurance affiliation. Noninclusion criteria were patients with untreated brain metastases, being pregnant or having contraindications to physical activity (e.g., uncontrolled hypertension, cardiac disease; unstable bone metastases); being unable to be followed for medical, social, family, geographic or psychological reasons over the study period or with deprivation of liberty by court or administrative decision. All patients provided written informed consent prior to inclusion in the study.

### Physical activity intervention

The intervention was a 6-month, home-based, unsupervised and personalized physical activity program based on the recommendations in terms of steps per day [41]. Patients were asked to wear an activity tracker during the duration of the intervention (i.e., 6 months). Based on their physical capacities evaluated at baseline and the average number of steps registered during the first week, women received an individual goal of steps per day from a physical activity instructor. The exercise goal was adapted weekly depending on the number of steps performed during the previous week and on the patient’s feelings, health and fitness status. All patients received weekly individual feedback from the physical activity instructor on their performance as well as personalized recommendations to increase or maintain their physical activity and reduce sedentary behaviour. The targeted number of steps was set within a maximum of 1000 steps above the average number of steps performed during the previous week. For participants reaching 10,000 steps per day, corresponding to the recommendations for healthy people [42], the goal was to maintain this number of steps.

### Data collection

#### Patients’ characteristics

Clinical data were extracted from the participants’ electronic medical records and included personal history of breast cancer, sites and number of metastases and current treatment. Anthropometric measurements taken at baseline and post-intervention, included standing height (cm), body weight (kg) and calculated Body Mass Index (BMI) (kg/m^2^). The quality of life was assessed with the European Organization for Research and Treatment of Cancer (EORTC) Quality of Life Questionnaire QLQ-C30 [43]. We used the global health status, functional status, physical function, symptoms and fatigue scores.

#### Physical activity fitness

The distance performed during the six-minute walk test [44] was determined as the maximum walking distance (6MWD) on a 30-m long flat corridor during six minutes. The isometric maximal strength of the dominant leg extensors was measured with a dynamometer (DFS II Series Digital Force Gauges, Chatillon, Florida, USA) attached at the ankle of the patient seated on a chair with the knee flexed at 90°; the highest value of two separate measures was registered. Hand grip strength was measured using a hand dynamometry (Jamar Plus Digital Hand Dynamometer, Patterson Medical, Huthwaite, United Kingdom) [45]; three measures were performed on each hand and the best performance was registered. The number of steps per day was measured using a connected activity tracker (Nokia Go® wristband, Nokia France, Issy-les-Moulineaux, France) and collected by regular transfer through a specific smartphone application (Nokia Health Mate®).

#### Physical activity level

Physical activity was evaluated by the French version of the long form of the International Physical Activity Questionnaire (IPAQ) [46], The long-form IPAQ is a validated self-administered physical activity questionnaire comprising 31 items grouped into four activity domains: work-related, transportation, domestic and recreational physical activity. The level of physical activity was determined by the IPAQ total physical activity score, expressed in metabolic equivalent of task (MET)-minutes per week. The total sitting time every date in the past week was estimated by asking the question “About how many hours in each 24 h-day do you usually spend on sitting?”.

#### Toxicities

Adverse events were extracted from the participants’ electronic medical records and were graded according to the toxicity criteria of the National Cancer Institute (Common Terminology Criteria for Adverse Events, CTCAE v5.0). The toxicity was defined as the occurrence of severe toxicity (grade > 2) throughout the study.

#### Computed tomography (CT)-based analyses of muscle

Skeletal muscle analyses based on the CT scans, were performed routinely in all patients with a metastatic diagnosis. We analysed only CT images performed within ± 30 days from baseline, three months and six months after study entry. A cross-sectional CT image at the midpoint of the third lumbar vertebrae (L3) was extracted from CT scan for each patient at each time using the Syngo.via software (Siemens Healthcare GmBH, Erlangen, Germany) and analysed using the National Institutes of Health (NIH) ImageJ software [47, 48]. The cross-sectional area (mm^2^) of the seven muscles of the L3 region (psoas, rector spinae, quadratus lumborum, transversus abdominus, external and internal obliques and rectus abdominus) was assessed by measuring the area composed by all pixels having an attenuation comprises between − 29 and + 150 Hounsfields Units (HU) excluding those located within internal cavity [36, 47, 49, 50]. Skeletal muscle density (SMD) (HU) represents the mean attenuation of these pixels. Skeletal muscle index (SMI) (cm^2^/m^2^) was obtained by normalizing cross-sectional muscle area by patient height. Skeletal muscle gauge (SMG) (HUxcm^2^/m^2^) was calculated multiplying muscle area by SMD. Skeletal muscle area at L3 was shown to predict whole lean body mass (LBM) according to the following equation [51]:

$${\text{LBM~}}\left( {kg} \right) = 0.3 \times cross~sec tional~muscle~area~at~L3~\,\left( {cm^{2} } \right) + 6.06$$

At baseline, patients were considered as sarcopenic if they had a SMI < 40cm^2^/m^2^ and patients were considered to have a poor muscle quality if they had a SMD < 37.8HU [52]. Sarcopenia and poor muscle quality are not mutually exclusive. Patients were classified as having sarcopenic obesity if they had a BMI > 30 kg/m^2^ and had sarcopenia.

#### Blood collection and oxidative stress analyses

A 7 mL blood sample was collected at baseline and six months. Blood samples were drawn into ethylenediaminetetraacetic acid (EDTA) blood collection tubes, centrifuged at 800 g during 10 min. Plasma and buffy coat were aliquoted into cryotubes, which were immediately frozen to − 80 °C until analysis.

All spectrophotometry and fluometry measurements were performed with TECAN Infinite 2000 plate reader (Männedorf, Switzerland). Plasma antioxidant enzymes (including catalase, glutathione peroxidase and superoxide dismutase activities), prooxidant enzymes (including NADPH oxidase activity and myeloperoxidase activities) and oxidative stress damage markers (including Advanced oxidation protein products [AOPP], Malondialdehyde [MDA] and DNA oxidation [8-Hydroxy-2′-deoxyguanosine; 8-OhdG] were measured as previously published [53, 54].

### Statistical analysis

**Fig. 1:**
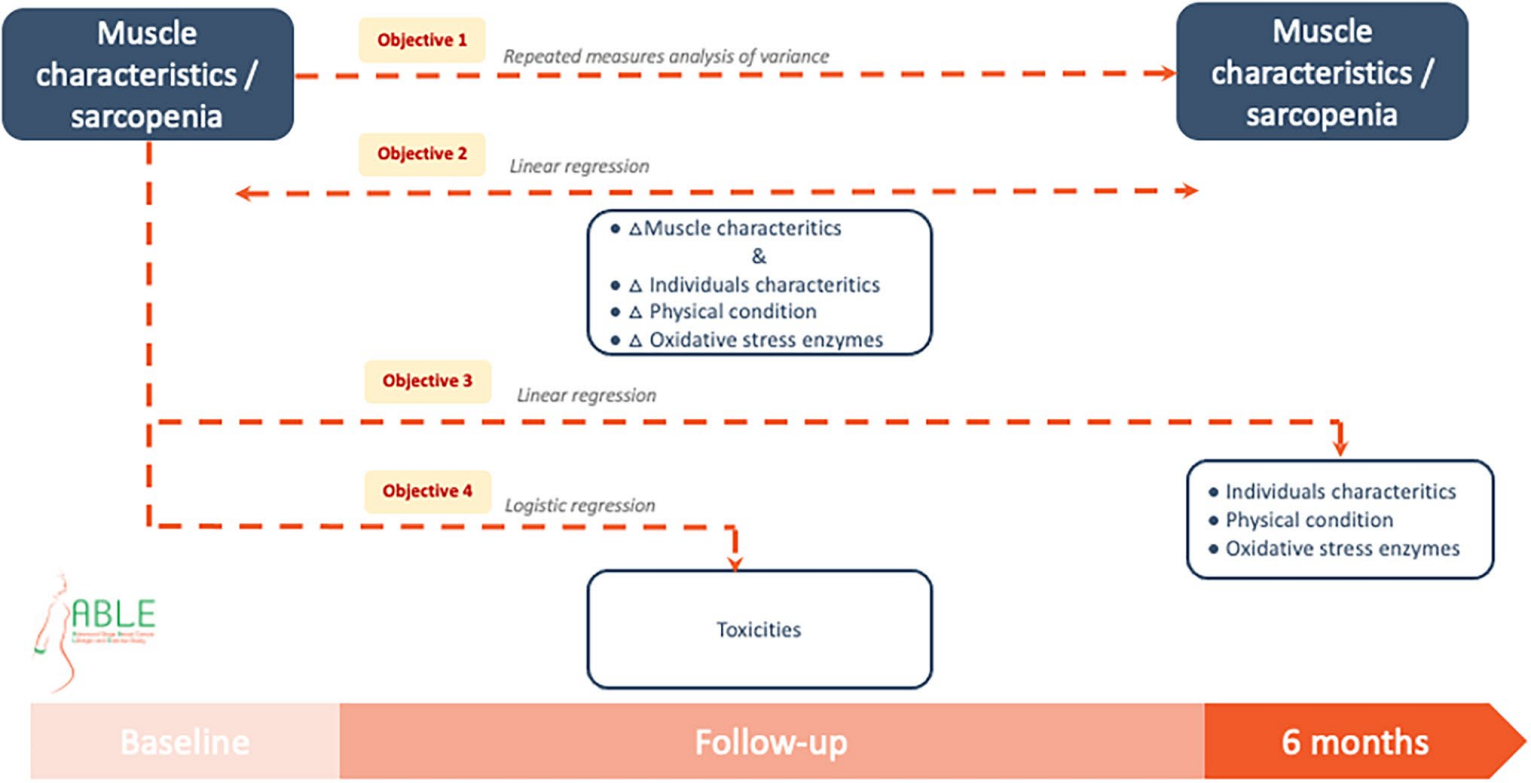
Statistical analysis from the ABLE study (*n* = 49)

Participants’ characteristics were described using means and standard deviations (SDs) or 95% confidence intervals (CIs) for quantitative data and frequencies and percentages for qualitative data. Boxplots were used to graphically display the distribution of the muscle variables (SMI, SMG, LBM, SMD and muscle cross-sectional area at L3). The changes in these muscle variable values and the sarcopenic status during the study was tested by repeated measures analyses of variance (Fig. 1). The associations between physical activity level, physical fitness, quality of life and the muscle’s characteristics were explored using linear regressions (Fig. 1). The association between sarcopenic status at baseline and the patients’ characteristics at the end of the intervention were explored using logistic regression (Fig. 1). Logistic regression was also used to explore the risk of severe toxicity associated with the sarcopenic status at baseline (Fig. 1).

Given the relatively small sample size, analyses were adjusted whenever possible and appropriate for age, BMI as continuous variable, and treatment (chemotherapy vs hormone therapy).

The data were analysed using the SAS software (version 9.4., SAS Institute Inc., Cary, NC, USA).

## Results

### Patients characteristics at baseline

Among the 49 metastatic breast cancer patients enrolled in the study (recruitment rate 94% of eligible patients), 47 had an available abdominal CT scan at baseline. At baseline, 25 (53.2%) patients were classified as sarcopenic, among whom three (12.0%) had a sarcopenic obesity (Table 1). A total of 35 patients (74.5%) had a poor muscle quality represented by a SMD < 37.8HU (Table 1): 16 among nonsarcopenic patients (72.7%) and 19 patients among sarcopenic patients (76%) had a poor muscle quality (SMD < 37.8HU) (Table 1). There were no statistically significant differences for age, height, SMD, number of metastatic localizations and type of treatment between nonsarcopenic and sarcopenic patients. However, patients with secondary metastatic breast cancer (*n* = 35, 71.4%) were more likely to be sarcopenic (*p* = 0.03) and had a statistically significantly lower BMI (*p* < 0.01), LBM (*p* < 0.01), SMI (*p* < 0.01) and SMG (*p* = 0.02) as compared to de novo metastatic breast cancer patients (supplementary data).

**Table 1:** Patient baseline characteristics from the ABLE study (*n* = 49)

	All patients (*n* = 49) Mean (SD) or *n* (%)
Age (year), mean (SD)	54.91 (10.41)
Anthropometrics	
Height (m), mean (SD)	162.76 (6.06)
Weight (kg), mean (SD)	69.12 (15.71)
BMI (kg/m^2^), mean (SD)	26.08 (5.78)
Underweight (< 18.5 kg/m^2^), *n* (%)	3 (6.1)
Normal weight (< 25 kg/m^2^), *n* (%)	20 (40.8)
Overweight (25–30 kg/m^2^), *n* (%)	16 (32.7)
Obese (> 30 kg/m^2^), *n* (%)	10 (20.4)
Muscle characteristics	
Cross-sectional area at L3 (cm^2^), mean (SD)	110.33 (16.42)
SMD (HU), mean (SD)	33.16 (8.89)
Low SMD (< 37.8HU)	35 (74.5)
SMI (cm^2^/m^2^), mean (SD)	41.72 (6.28)
LBM (kg), mean (SD)	39.16 (4.93)
SMG (arbitrary units), mean (SD)	1389.95 (458.03)
Sarcopenia, *n* (%)	25 (53.2)
Clinical	
Number of metastatic localizations, *n* (%)	4.65 (3.05)
De novo metastatic breast cancer, *n* (%)	14 (28.6)
Hormone therapy, *n* (%)^a^	30 (61.2)
Chemotherapy, *n* (%)^a^	25 (51.0)
Toxicities	
Number of toxicities (≥ grade 3)	19 (38.8)
Types of toxicities	
Hematologic	6 (24.0)
Metabolic	3 (12.0)
Neurological	3 (12.0)
Pneumological	3 (12.0)
Gastrointestinal	2 (8.0)
Cutaneous	2 (8.0)
Vascular	1 (4.0)
Fatigue	1 (4.0)
Infection	1 (4.0)
Musculoskeletal	1 (4.0)
Asthenia	1 (4.0)
General worsening	1 (4.0)

*SMD* skeletal muscle density, *SMI* skeletal muscle index, *LBM* lean body mass, *SMG* skeletal muscle gauge, *BMI* Body Mass Index

^a^*n* = 6 patients received concomitant chemotherapy and hormone therapy

### Evolution of physical fitness and muscle characteristics during the physical activity intervention

Overall, there was a statistically significant improvement in the 6-minute walking distance (6MWD, + 7%, *p* < 0.001) and the isometric quadriceps strength (+ 22%, *p* < 0.001) between baseline and the end of the study [55].

**Fig. 2:**
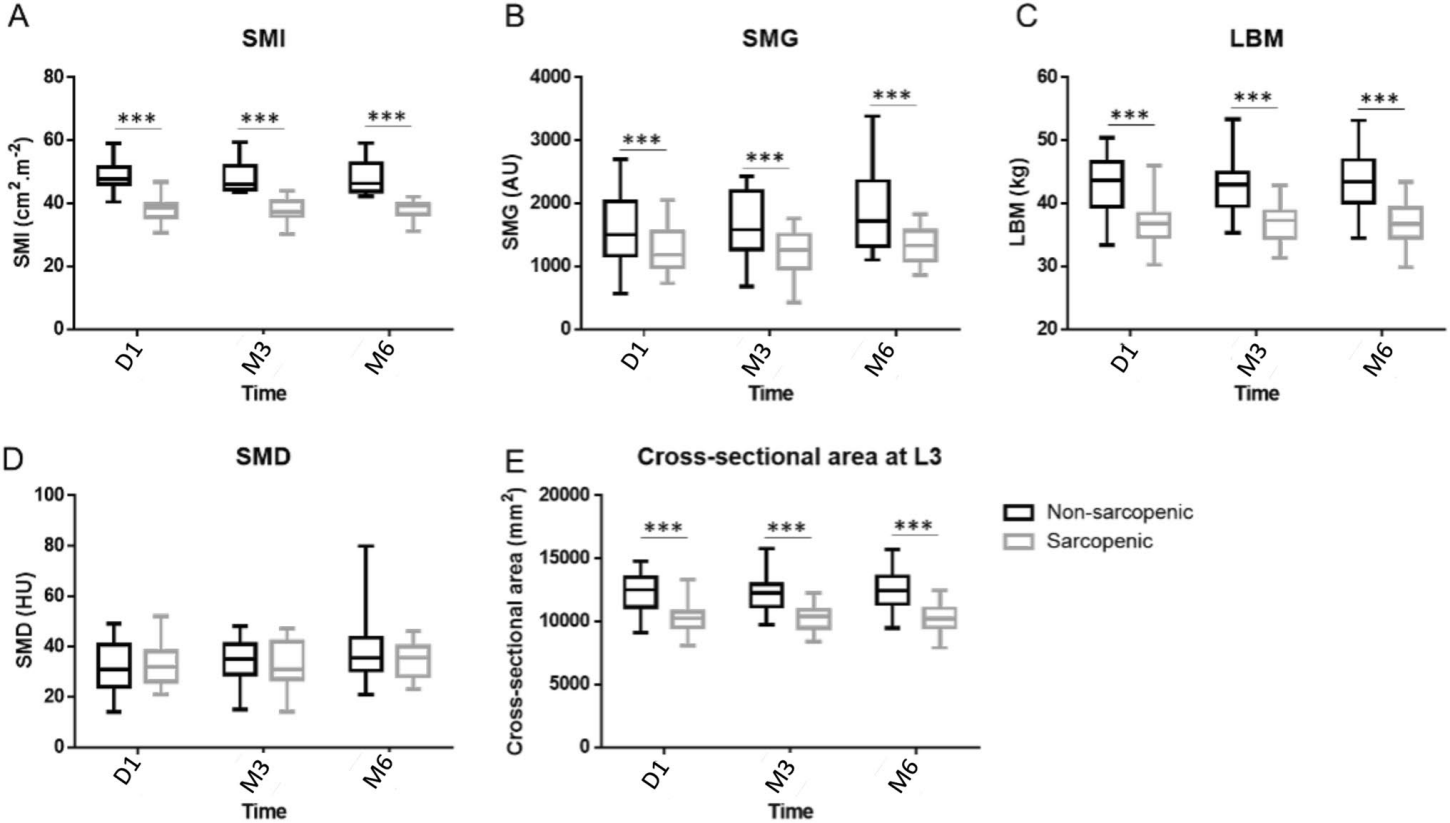
Variation of SMI (**a**), SMG (**b**), LBM (**c**), SMD (**d**) and muscle cross-sectional area at L3 at three points of the study (baseline, 3 months, 6 months) according to sarcopenic status from the ABLE study (*n* = 49). ***p < 0.001

However, cross sectional muscle area, skeletal muscle radiodensity, LBM and skeletal muscle gauge remained constant over the 6 months (*p* = 0.75, *p* = 0.07, *p* = 0.75 and *p* = 0.06, respectively), but differed significantly between sarcopenic and nonsarcopenic patients at baseline, three months and six months (Fig. 2).

### Variation between baseline and 6 months in muscle characteristics and physical condition and quality of life

No difference in physical performance was observed between sarcopenic and nonsarcopenic participants at baseline except for isometric quadriceps strength (227 N vs 170 N, *p* = 0.01). Moreover, the number of daily steps (6038 vs 4009, *p* = 0.05) as well as the total level of physical activity (2309 vs 1854 MET-minutes/week, *p* = 0.28) was higher in sarcopenic as compared to nonsarcopenic participants.

Regarding the associations between baseline and six months in muscle characteristics and physical activity level, physical fitness, quality of life after adjusting on age and BMI, an association was shown for the SMG (*p* = 0.03) and the SMD with the reduction of sitting time (*p* = 0.04) between baseline and the end of the intervention (Table 2).

**Table 2:** Variation between baseline and month six in muscle characteristics and independent variables, adjusted on age and BMI from the ABLE study (*n* = 49)

Muscle characteristics				
Independent variables	Cross-sectional area parameter (SD), *p* value)	SMI (parameter (SD), *p* value)	SMG parameter (SD), *p* value)	SMD parameter (SD), *p* value)
Physical activity level				
Total physical activity (MET-minutes/week)	− 0.02 (0.06) 0.75	− < 0.01 (< 0.01) 0.85	0.02 (0.04) 0.64	< 0.01 (< 0.01) 0.62
Moderate physical activity (MET-minutes/week)	− 0.01 (0.08) 0.87	− < 0.01 (< 0.01) 0.98	0.02 (0.05) 0.71	< 0.01 (< 0.01) 0.75
Vigorous physical activity (MET-minutes/week)	− 0.49 (0.78) 0.53	− < 0.01 (< 0.01) 0.65	− 0.11 (0.51) 0.83	− 0.01 (0.01) 0.87
Sitting time (minutes/week)	0.09 (0.11) 0.41	< 0.01 (< 0.01) 0.46	0.16 (0.07) **0.03**	0.01 (< 0.01) **0.04**
Steps/day	0.05 (0.08) 0.56	< 0.01 (< 0.01) 0.56	− 0.03 (0.03) 0.34	− < 0.01 (< 0.01) 0.25
Physical fitness				
6 min walking test (m)	− 2.60 (1.70) 0.14	− 0.01(< 0.01) 0.15	− 2.07 (1.05) 0.06	− 0.04 (0.02) 0.08
Handgrip strength right (kg)	40.55 (33.44) 0.23	0.15 (0.13) 0.25	20.17 (22.16) 0.35	0.29 (0.50) 0.57
Handgrip strength left (kg)	0.04 (3.55) 0.99	< 0.01 (0.01) 0.98	3.47 (2.27) 0.14	0.08 (0.05) 0.11
Isometric quadriceps strength (N)	2.11 (2.39) 0.38	0.01 (< 0.01) 0.33	0.85 (1.55) 0.59	0.01 (0.04) 0.74
Quality of life				
Global health	− 2.85 (6.03) 0.64	− 0.01 (0.02) 0.73	2.00 (3.87) 0.61	0.04 (0.09) 0.65
Physical functioning	− 3.86 (7.33) 0.60	− 0.01 (0.03) 0.66	− 2.78 (4.71) 0.56	− 0.04 (0.11) 0.64
Fatigue symptom	2.30 (5.60) 0.68	0.01 (0.02) 0.72	5.03 (3.51) 0.16	0.11 (0.08) 0.18

Independent variables: physical activity level, physical fitness, quality of life

*SMD* skeletal muscle density, *SMI* Skeletal Muscle Index, *SMG* skeletal muscle gauge

*p* value are obtained using beta regression coefficients

### Association between sarcopenia at baseline and individual characteristics, physical activity condition and oxidative stress at 6 months

Being sarcopenic at baseline was associated with a lower BMI, cross sectional area at L3, SMG, SMI and LBM at the end of the 6-month intervention study (*p* < 0.01, *p* < 0.01, *p* = 0.04, *p* < 0.01 and *p* < 0.01, respectively) (Table 3). In addition, although not significant, the total level of physical activity at 6 months was lower in sarcopenic participants compared to nonsarcopenic participants (1728 vs 2140 MET-minutes/week, *p* = 0.74) in contrast to the baseline data.

**Table 3:** Associations between sarcopenia at baseline, individual characteristics, physical condition and oxidative stress enzymes at 6 months from the ABLE study (*n* = 49)

Month 6	All patients (*n* = 47) Mean (SD) or *n* (%)	Nonsarcopenic (*n* = 22) Mean (SD) or *n* (%)	Sarcopenic (*n* = 25) Mean (SD) or *n* (%)	*p* value
Anthropometrics				
Weight (kg), mean (SD)	67.37 (15.59)	75.21 (17.26)	61.17 (10.96)	** < 0.01**
BMI (kg/m^2^), mean (SD)	25.41 (5.91)	28.68 (6.55)	22.81 (3.77)	** < 0.01**
Underweight (< 18.5 kg/m^2^), *n* (%)	3.0 (7.0)	1.0 (5.3)	2.0 (8.3)	**0.02**
Normal weight (< 25 kg/m^2^), *n* (%)	21.0 (48.8)	5.0 (26.3)	16.0 (66.7)	
Overweight (25–30 kg/m^2^), *n* (%)	11.0 (25.6)	6.0 (31.6)	5.0 (20.8)	
Obese (> 30 kg/m^2^), *n* (%)	8.0 (18.6)	7.0 (36.8)	1.0 (4.2)	
Muscle characteristics				
Cross-sectional area at L3 (cm^2^), mean (SD)	110.19 (16.88)	121.03 (18.65)	102.06 (9.50)	** < 0.01**
SMG (arbitrary units), mean (SD)	1721.00 (619.03)	1328.11 (321.71)	1496.49 (505.64)	**0.04**
Low SMD (< 37.8HU)	26.0 (61.9)	12.0 (66.7)	14.0 (58.3)	0.58
SMI (cm^2^/m^2^), mean (SD)	41.46 (6.19)	45.96 (6.66)	38.08 (2.78)	** < 0.01**
LBM (kg), mean (SD)	39.12 (5.07)	42.37 (5.59)	36.68 (2.85)	** < 0.01**
SMD (HU), mean (SD)	35.99 (10.36)	37.61 (13.29)	34.78 (7.56)	0.84
Physical fitness				
6 min walking distance (m), mean (SD)	481.44 (107.23)	450.00 (114.38)	506.33 (96.42)	0.10
Handgrip strength right (kg), mean (SD)	26.21 (4.34)	25.67 (4.75)	26.66 (4.02)	0.65
Handgrip strength left (kg), mean (SD)	24.04 (4.44)	24.13 (4.39)	23.98 (4.57)	0.87
Isometric quadriceps strength (N), mean (SD)	239.19 (77.28)	259.60 (81.57)	223.87 (71.83)	0.16
Physical activity level				
Total physical activity (MET-minutes/week)	1910.19 (1771.40)	2140.32 (2275.71)	1728.00 (1264.36)	0.74
Sitting time (min/week)	1728.60 (846.98)	1554.74 (897.94)	1866.25 (796.38)	0.18
Mean steps per day over a month, mean (SD)	5266.91 (2736.79)	4475.36 (2889.51)	6011.90 (2436.46)	0.08
Antioxidant enzymes				
CAT (µmol/min/L), mean (SD)	37.83 (18.92)	35.51 (14.89)	39.67 (21.73)	0.73
GPx (mol/min/L), mean (SD)	82.05 (25.63)	82.59 (24.87)	81.64 (26.70)	0.94
SOD (mol/min/L), mean (SD)	8.88 (4.57)	10.52 (4.74)	7.57 (4.07)	0.06
Pro-oxidant enzymes				
NADPH oxidase leucocytes, mean (SD)	0.22 (0.06)	0.22 (0.06)	0.22 (0.06)	0.66
MPO Plasma, mean (SD)	119.27 (49.38)	131.87 (52.10)	109.30 (45.76)	0.08
MPO Leuco, mean (SD)	15.73 (7.95)	15.51 (6.67)	15.91 (8.97)	0.92
End products of OS damage lipids				
MDA (µmol/L), mean (SD)	12.53 (3.54)	11.21 (2.22)	13.57 (4.05)	**0.02**
DNA				
8-OHdG (µg/L), mean (SD)	18.00 (15.49)	16.77 (15.20)	18.97 (15.98)	0.66
Protein				
AOPP (µmol/L), mean (SD)	88.23 (56.25)	86.65 (53.01)	89.49 (59.79)	0.85
Inflammation				
IL6 (µmol/L), mean (SD)	24.41 (9.41)	35.55 (13.28)	33.50 (4.64)	0.79

*SMD* skeletal muscle density, *SMI* Skeletal Muscle Index, *LBM* lean body mass, *SMG* skeletal muscle gauge, *BMI* Body Mass Index, *CAT* catalase, *GPx* glutathione peroxidase, *SOD* superoxide dismutase, *MPO* myeloperoxidase, *MDA* malondialdehyde, *8-OHdG* 8-hydroxydésoxyguanosine, *AOPP* advanced oxidative protein products, *IL* interleukin

With respect to the biological markers, the sarcopenic status at baseline was associated with a higher plasma MDA concentration at 6 months (*p* = 0.02) (Table 3). The sarcopenic status at baseline was not associated with other oxidative stress biomarkers.

### Association between sarcopenia at baseline and the risk of toxicities during the 6-month physical activity intervention

A total of 41 patients experienced toxicities during the study, ranging from grade 1 to grade 5, including 19 severe toxicities (grade ≥ 3 (Table 1)). Being sarcopenic during the study was significantly associated with a higher risk of severe toxicity (≥ grade 3) during the six-month study (OR 4.56, 95% CI 1.07–19.41) after adjusting for age and chemotherapy.

## Discussion

This ancillary study in the ABLE Trial is the first study worldwide to analyse the evolution of sarcopenia during a six-month physical activity intervention in patients with metastatic breast cancer. One of its major findings is the maintenance of muscle mass during the 6-month intervention despite possible adverse effects of treatments and disease progression.

The high prevalence of sarcopenia and poor muscle quality at baseline in the ABLE Trial is consistent with the literature that has shown an increasing prevalence of sarcopenia with cancer stage. Several mechanisms may be involved, such as cancer and treatment-related metabolic disturbances, degeneration of satellite cells associated with aging, decreased physical activity and decreased dietary intake [56–58]. The combination of these factors acts on the balance of the muscle mass regulation and can cause an imbalance between the synthesis and degradation of muscle proteins in favour of degradation [59], leading to muscle atrophy [60–62]. A study of 3241 patients with localized breast cancer found that 34% were sarcopenic and 37% had poor muscle quality [52], in contrast with a study of 166 patients with metastatic breast cancer among whom 67% were sarcopenic and 60% had low muscle mass [63]. These observations in metastatic breast cancer are consistent with the skeletal muscle measures in the ABLE Trial with 53% women being sarcopenic and 75% having a low SMD. Furthermore, the results of the ABLE study do not show differences in muscle quality between sarcopenic and nonsarcopenic patients. In both groups, lipid infiltration was below the threshold (< 37.8 HU) raising the need to measure both the sarcopenia indicator and the muscle quality indicator, both of which are correlated with an increased risk of death [9]. Using a threshold for SMI of ≤ 41cm^2^/m^2^ as proposed by Martin et al., another cross-sectional study in 41 metastatic breast cancer patients reported a 34% prevalence of sarcopenia [64, 65]. These results reinforce the need to systematically assess body composition and sarcopenia in patients with cancer. However, additional studies are needed to determine valid thresholds for defining sarcopenia, making comparisons between studies easier [30, 66–68].

In the ABLE Trial, we previously found that the intervention improved functional capacities (i.e., statistically significant and clinically relevant improvements of 6MWD + 7% [69–71], isometric quadriceps strength + 22%) and that muscular strength was maintained despite the progress of the disease and treatment [55]. To our knowledge, only one randomized controlled trial conducted among 200 patients with localized breast cancer receiving adjuvant chemotherapy has assessed the impact of resistance and aerobic exercise on sarcopenia [35]. After a 17-week intervention, the arm doing resistance exercise had a statistically higher SMI as compared to the control group, suggesting a potential effect of a resistance exercise to reverse sarcopenic status [72]. Conversely, during the ABLE Trial the prevalence of sarcopenia and the skeletal muscle parameters remained constant at 3 and 6 months which suggests that even regular walking could preserve muscle mass. The reduction (by 5.0 ± 2.5 cm^2^ per year) of muscle mass or muscle quality observed in patients with a metastatic breast cancer [73, 74] underlines the potential importance of the suggested benefit of the six-month physical activity intervention of the ABLE Trial to mitigate treatment and disease induced sarcopenia in advanced breast cancer. There is increasing evidence that physical inactivity contributes to increase the prevalence of sarcopenia, particularly in the elderly [4]. The observed significant association of muscle density and muscle gauge with decreased sitting time in the ABLE trial, further suggests a beneficial effect of reducing sedentary behaviour in advanced breast cancer patients and may have in turn a beneficial effect on quality of life and survival [8, 10]. Although physical activity can sometimes seem complicated in this population, limiting physical inactivity can be a first step to achieving early clinical benefits. In this study, sarcopenic patients achieved a significant higher number of daily steps and total physical activity level as compared to nonsarcopenic participants. It is possible that patients awareness of the negative effects of lack of physical activity might have been an incentive to be more physically active. Drivers for change in behaviour and motivation related to physical activity in this population should be investigated in future studies.

Cancer and chemotherapy related enhanced production of reactive oxygen species is thought to promote sarcopenia, contribute to apoptotic changes in skeletal muscle fibres and alter the contractile qualities of the muscle through several molecular signalling pathways involved in the regulation of muscle tissue [75, 76]. In the present study, no significant association of sarcopenic status at baseline was observed with biomarkers of oxidative stress, except for MDA plasma levels at 6 months. MDA a common measure of oxidative stress, is produced at high levels during lipid peroxidation and has been shown to be involved in age-related diseases including cancer [75]. The observed association of sarcopenia with high plasma MDA at 6-month is consistent with increased MDA levels in sarcopenic elderly compared to their nonsarcopenic counterparts [38, 77]. Furthermore, plasma MDA has been suggested as a biomarker of cancer and tumour progression [78–80]. Also, a significant increase was observed in patients undergoing treatment for cervical cancer as compared to pretreatment levels [81]. An inverse relationship of MDA concentrations with the amount of physical activity in elderly women has been reported, and exercise has been suggested to decrease MDA plasma levels in a recent study including 130 non metastatic breast cancer patients [82].

In the ABLE Trial, the incidence of severe toxicity (40%) was higher than in patients with localized breast cancer (19%) [72] but lower compared to observed prevalence in two other studies of 40 and 66 metastatic breast cancer patients reporting grade 3–4 toxicity rates in 55% and 57% of sarcopenic patients, respectively [10]. Grade 3–4 toxicity rates in nonsarcopenic patients in these studies (20% and 18% respectively were similar to our study. The results of this study further confirm a link between sarcopenia and treatment toxicities consistent with the literature showing an increased risk of toxicity in sarcopenic patients as well as a correlation of low SMI and muscle area with severe toxicities [72]. It has been suggested that the use of lean muscle mass instead of body surface area to calculate chemotherapy dose could limit the risk of toxicity and treatment delay as well as improve chemotherapy relative dose intensity [19]. This may in turn improve treatment efficacy while improving patients’ quality of life [12, 16, 19, 30, 83]. The CT scan-based analyses of the skeletal muscle area on cross section at the third lumbar vertebra (L3) provides a reliable representation of the total body muscle mass and has therefore been widely adopted for the detection of sarcopenia in cancer patients [36, 51].

The strengths of this ancillary analysis of ABLE Trial were the high recruitment rate, low attrition, excellent adherence to the physical activity intervention and repeated CT scan to monitor sarcopenia over time. The limitations of this single-center study include the lack of a control group, the small sample size that limits statistical power and, finally, the short follow-up that did not allow survival analyses to be carried out according to sarcopenic status. Many of the tests were done without adjustment given the exploratory nature of the study.

## Conclusion

This ancillary analysis of the ABLE Trial provides important preliminary data on potential benefits of physical activity in preventing sarcopenia and chemotoxicities in patients with metastatic breast cancer. The emergence of artificial intelligence methods to assess body composition and sarcopenia in routine will lead to considerable progress in patient care [84]. This will facilitate the use of lean muscle mass to calculate chemotherapy dose to reduce toxicity in sarcopenic patients in future studies. Also, clinicians should encourage women with metastatic breast cancer to remain physically active and limit sedentarity [85]. Finally, these preliminary data provide a strong rational for the development of a larger multicentre randomized trial to evaluate the impact of a physical intervention specifically on the muscle mass and chemotherapy toxicities.

## Supplementary Information

Below is the link to the electronic supplementary material.

Supplementary file1 (DOCX 17 kb)

## Abbreviations

BMI: Body Mass Index
MET: Metabolic equivalent of task
6MWD: Six-minute walking distance

## References

[1] Cardoso F, Costa A, Senkus E (2017). 3rd ESO-ESMO International Consensus Guidelines for Advanced Breast Cancer (ABC 3). Ann Oncol.

[2] O’Shaughnessy J (2005). Extending survival with chemotherapy in metastatic breast cancer. Oncologist.

[3] Diaby V, Tawk R, Sanogo V (2015). A review of systematic reviews of the cost-effectiveness of hormone therapy, chemotherapy, and targeted therapy for breast cancer. Breast Cancer Res Treat.

[4] Cruz-Jentoft AJ, Baeyens JP, Bauer JM (2010). Sarcopenia: European consensus on definition and diagnosis: report of the European Working Group on Sarcopenia in Older People. Age Ageing.

[5] Kiss N, Loeliger J, Findlay M (2020). Clinical Oncology Society of Australia: position statement on cancer-related malnutrition and sarcopenia. Nutr Diet.

[6] Cruz-Jentoft AJ, Bahat G, Bauer J (2019). Sarcopenia: revised European consensus on definition and diagnosis. Age Ageing.

[7] Shachar SS, Williams GR, Muss HB, Nishijima TF (2016). Prognostic value of sarcopenia in adults with solid tumours: a meta-analysis and systematic review. Eur J Cancer.

[8] Zhang X-M, Dou Q-L, Zeng Y (2020). Sarcopenia as a predictor of mortality in women with breast cancer: a meta-analysis and systematic review. BMC Cancer.

[9] Rier HN, Jager A, Sleijfer S (2017). Low muscle attenuation is a prognostic factor for survival in metastatic breast cancer patients treated with first line palliative chemotherapy. Breast.

[10] Shachar SS, Deal AM, Weinberg M (2017). Skeletal muscle measures as predictors of toxicity, hospitalization, and survival in patients with metastatic breast cancer receiving taxane-based chemotherapy. Clin Cancer Res.

[11] Versteeg KS, Blauwhoff-Buskermolen S, Buffart LM (2018). Higher muscle strength is associated with prolonged survival in older patients with advanced cancer. Oncologist.

[12] Prado CMM, Baracos VE, McCargar LJ (2009). Sarcopenia as a determinant of chemotherapy toxicity and time to tumor progression in metastatic breast cancer patients receiving capecitabine treatment. Clin Cancer Res.

[13] Franzoi MA, Vandeputte C, Eiger D (2020). Computed tomography-based analyses of baseline body composition parameters and changes in breast cancer patients under treatment with CDK 4/6 inhibitors. Breast Cancer Res Treat.

[14] Aubrey J, Esfandiari N, Baracos VE (2014). Measurement of skeletal muscle radiation attenuation and basis of its biological variation. Acta Physiol (Oxf).

[15] Goodpaster BH, Chomentowski P, Ward BK (2008). Effects of physical activity on strength and skeletal muscle fat infiltration in older adults: a randomized controlled trial. J Appl Physiol.

[16] Prado CMM, Baracos VE, McCargar LJ (2007). Body composition as an independent determinant of 5-fluorouracil-based chemotherapy toxicity. Clin Cancer Res.

[17] Prado CMM, Lima ISF, Baracos VE (2011). An exploratory study of body composition as a determinant of epirubicin pharmacokinetics and toxicity. Cancer Chemother Pharmacol.

[18] Iwase T, Sangai T, Nagashima T (2016). Impact of body fat distribution on neoadjuvant chemotherapy outcomes in advanced breast cancer patients. Cancer Med.

[19] Barret M, Antoun S, Dalban C (2014). Sarcopenia is linked to treatment toxicity in patients with metastatic colorectal cancer. Nutr Cancer.

[20] Antoun S, Baracos VE, Birdsell L (2010). Low body mass index and sarcopenia associated with dose-limiting toxicity of sorafenib in patients with renal cell carcinoma. Ann Oncol.

[21] Massicotte M-H, Borget I, Broutin S (2013). Body composition variation and impact of low skeletal muscle mass in patients with advanced medullary thyroid carcinoma treated with vandetanib: results from a placebo-controlled study. J Clin Endocrinol Metab.

[22] Aleixo GFP, Williams GR, Nyrop KA (2019). Muscle composition and outcomes in patients with breast cancer: meta-analysis and systematic review. Breast Cancer Res Treat.

[23] Villaseñor A, Ballard-Barbash R, Baumgartner K (2012). Prevalence and prognostic effect of sarcopenia in breast cancer survivors: the HEAL Study. J Cancer Surviv.

[24] Del Fabbro E, Parsons H, Warneke CL (2012). The relationship between body composition and response to neoadjuvant chemotherapy in women with operable breast cancer. Oncologist.

[25] Wong AL, Seng KY, Ong EM (2014). Body fat composition impacts the hematologic toxicities and pharmacokinetics of doxorubicin in Asian breast cancer patients. Breast Cancer Res Treat.

[26] Shachar SS, Deal AM, Weinberg M (2017). Body composition as a predictor of toxicity in patients receiving anthracycline and taxane-based chemotherapy for early-stage breast cancer. Clin Cancer Res.

[27] Huillard O, Mir O, Peyromaure M (2013). Sarcopenia and body mass index predict sunitinib-induced early dose-limiting toxicities in renal cancer patients. Br J Cancer.

[28] Mir O, Coriat R, Blanchet B (2012). Sarcopenia predicts early dose-limiting toxicities and pharmacokinetics of sorafenib in patients with hepatocellular carcinoma. PLoS ONE.

[29] Du Bois D, Du Bois EF (1989). A formula to estimate the approximate surface area if height and weight be known. 1916. Nutrition.

[30] Prado CMM, Lieffers JR, McCargar LJ (2008). Prevalence and clinical implications of sarcopenic obesity in patients with solid tumours of the respiratory and gastrointestinal tracts: a population-based study. Lancet Oncol.

[31] Antoun S, Borget I, Lanoy E (2013). Impact of sarcopenia on the prognosis and treatment toxicities in patients diagnosed with cancer. Curr Opin Support Palliat Care.

[32] Jung H-W, Kim JW, Kim J-Y (2015). Effect of muscle mass on toxicity and survival in patients with colon cancer undergoing adjuvant chemotherapy. Support Care Cancer.

[33] Courneya KS, Segal RJ, Mackey JR (2007). Effects of aerobic and resistance exercise in breast cancer patients receiving adjuvant chemotherapy: a multicenter randomized controlled trial. J Clin Oncol.

[34] Speck RM, Courneya KS, Mâsse LC (2010). An update of controlled physical activity trials in cancer survivors: a systematic review and meta-analysis. J Cancer Surviv.

[35] Adams SC, Segal RJ, McKenzie DC (2016). Impact of resistance and aerobic exercise on sarcopenia and dynapenia in breast cancer patients receiving adjuvant chemotherapy: a multicenter randomized controlled trial. Breast Cancer Res Treat.

[36] Shen W, Punyanitya M, Wang Z (2004). Total body skeletal muscle and adipose tissue volumes: estimation from a single abdominal cross-sectional image. J Appl Physiol.

[37] Can B, Kara O, Kizilarslanoglu MC (2017). Serum markers of inflammation and oxidative stress in sarcopenia. Aging Clin Exp Res.

[38] Coto Montes A, Boga JA, Bermejo Millo C (2017). Potential early biomarkers of sarcopenia among independent older adults. Maturitas.

[39] Puig-Vilanova E, Rodriguez DA, Lloreta J (2015). Oxidative stress, redox signaling pathways, and autophagy in cachectic muscles of male patients with advanced COPD and lung cancer. Free Radical Biol Med.

[40] Sullivan-Gunn MJ, Lewandowski PA (2013). Elevated hydrogen peroxide and decreased catalase and glutathione peroxidase protection are associated with aging sarcopenia. BMC Geriatr.

[41] Delrieu L, Pérol O, Fervers B (2018). A Personalized physical activity program with activity trackers and a mobile phone app for patients with metastatic breast cancer: protocol for a single-arm feasibility trial. JMIR Res Protocols.

[42] Tudor-Locke C, Hatano Y, Pangrazi RP, Kang M (2008). Revisiting “how many steps are enough?”. Med Sci Sports Exerc.

[43] Hjermstad MJ, Fossa SD, Bjordal K, Kaasa S (1995). Test/retest study of the European Organization for Research and Treatment of Cancer Core Quality-of-Life Questionnaire. J Clin Oncol.

[44] Galiano-Castillo N, Arroyo-Morales M, Ariza-Garcia A (2016). The six-minute walk test as a measure of health in breast cancer patients. J Aging Phys Act.

[45] Savva C, Karagiannis C, Rushton A (2013). Test–retest reliability of grip strength measurement in full elbow extension to evaluate maximum grip strength. J Hand Surg Eur.

[46] Crinière L, Lhommet C, Caille A (2011). Reproducibility and validity of the French version of the long international physical activity questionnaire in patients with type 2 diabetes. J Phys Act Health.

[47] Gomez-Perez SL, Haus JM, Sheean P (2016). Measuring abdominal circumference and skeletal muscle from a single cross-sectional computed tomography image: a step-by-step guide for clinicians using National Institutes of Health ImageJ. J Parenter Enter Nutr.

[48] Schneider CA, Rasband WS, Eliceiri KW (2012). NIH Image to ImageJ: 25 years of image analysis. Nat Methods.

[49] McLean RR, Kiel DP (2015). Developing consensus criteria for sarcopenia: an update. J Bone Miner Res.

[50] Mitsiopoulos N, Baumgartner RN, Heymsfield SB (1998). Cadaver validation of skeletal muscle measurement by magnetic resonance imaging and computerized tomography. J Appl Physiol.

[51] Mourtzakis M, Prado CMM, Lieffers JR (2008). A practical and precise approach to quantification of body composition in cancer patients using computed tomography images acquired during routine care. Appl Physiol Nutr Metab.

[52] Caan BJ, Cespedes Feliciano EM, Prado CM (2018). Association of muscle and adiposity measured by computed tomography with survival in patients with nonmetastatic breast cancer. JAMA Oncol.

[53] Faiss R, Pialoux V, Sartori C (2013). Ventilation, oxidative stress, and nitric oxide in hypobaric versus normobaric hypoxia. Med Sci Sports Exerc.

[54] Ribon-Demars A, Pialoux V, Boreau A et al (2018) Protective roles of estradiol against vascular oxidative stress in ovariectomized female rats exposed to normoxia or intermittent hypoxia. Acta Physiol (Oxf). 10.1111/apha.13159 10.1111/apha.13159 29947475

[55] Delrieu L, Pialoux V, Pérol O (2020). Feasibility and Health benefits of an individualized physical activity intervention in women with metastatic breast cancer: intervention Study. JMIR Mhealth Uhealth.

[56] Jeejeebhoy KN (2012). Malnutrition, fatigue, frailty, vulnerability, sarcopenia and cachexia: overlap of clinical features. Curr Opin Clin Nutr Metab Care.

[57] Kim TN, Choi KM (2013). Sarcopenia: definition, epidemiology, and pathophysiology. J Bone Metab.

[58] Rolland Y, Czerwinski S, Abellan Van Kan G (2008). Sarcopenia: its assessment, etiology, pathogenesis, consequences and future perspectives. J Nutr Health Aging.

[59] Tsai S (2012). Importance of lean body mass in the oncologic patient. Nutr Clin Pract.

[60] Kazemi-Bajestani SMR, Becher H, Fassbender K (2014). Concurrent evolution of cancer cachexia and heart failure: bilateral effects exist. J Cachexia Sarcopenia Muscle.

[61] Brioche T, Lemoine-Morel S (2016). Oxidative stress, sarcopenia, antioxidant strategies and exercise: molecular aspects. Curr Pharm Des.

[62] Prado CMM, Lieffers JR, Bowthorpe L (2013). Sarcopenia and physical function in overweight patients with advanced cancer. Can J Diet Pract Res.

[63] Rier HN, Jager A, Sleijfer S (2016). The prevalence and prognostic value of low muscle mass in cancer patients: a review of the literature. Oncologist.

[64] Sheean P, Gomez-Perez S, Joyce C (2019). Body composition, serum biomarkers of inflammation and quality of life in clinically stable women with estrogen receptor positive metastatic breast cancer. Nutr Cancer.

[65] Martin L, Birdsell L, MacDonald N (2013). Cancer cachexia in the age of obesity: skeletal muscle depletion is a powerful prognostic factor, independent of Body Mass Index. JCO.

[66] Fearon K, Strasser F, Anker SD (2011). Definition and classification of cancer cachexia: an international consensus. Lancet Oncol.

[67] Martin L, Birdsell L, Macdonald N (2013). Cancer cachexia in the age of obesity: skeletal muscle depletion is a powerful prognostic factor, independent of body mass index. J Clin Oncol.

[68] Sandini M, Bernasconi DP, Fior D (2016). A high visceral adipose tissue-to-skeletal muscle ratio as a determinant of major complications after pancreatoduodenectomy for cancer. Nutrition.

[69] Bohannon RW, Crouch R (2017). Minimal clinically important difference for change in 6-minute walk test distance of adults with pathology: a systematic review: Systematic review of MCID in 6MWT. J Eval Clin Pract.

[70] Schmidt K, Vogt L, Thiel C (2013). Validity of the six-minute walk test in cancer patients. Int J Sports Med.

[71] Solway S, Brooks D, Lacasse Y, Thomas S (2001). A qualitative systematic overview of the measurement properties of functional walk tests used in the cardiorespiratory domain. Chest.

[72] Mazzuca F, Onesti CE, Roberto M (2018). Lean body mass wasting and toxicity in early breast cancer patients receiving anthracyclines. Oncotarget.

[73] Solomayer E-F, Braun E-M, Zimmermann JSM (2019). Muscle mass loss in patients with metastatic breast cancer. Arch Gynecol Obstet.

[74] Rier HN, Jager A, Sleijfer S (2018). Changes in body composition and muscle attenuation during taxane-based chemotherapy in patients with metastatic breast cancer. Breast Cancer Res Treat.

[75] Ábrigo J, Elorza AA, Riedel CA (2018). Role of oxidative stress as key regulator of muscle wasting during cachexia. Oxid Med Cell Longev.

[76] Peixoto da Silva S, Santos JMO, Costa e Silva MP (2020). Cancer cachexia and its pathophysiology: links with sarcopenia, anorexia and asthenia. J Cachexia Sarcopenia Muscle.

[77] Bellanti F, Romano AD, Lo Buglio A (2018). Oxidative stress is increased in sarcopenia and associated with cardiovascular disease risk in sarcopenic obesity. Maturitas.

[78] Kundaktepe BP, Sozer V, Durmus S (2021). The evaluation of oxidative stress parameters in breast and colon cancer. Medicine (Baltimore).

[79] Jelic MD, Mandic AD, Maricic SM, Srdjenovic BU (2021). Oxidative stress and its role in cancer. J Cancer Res Ther.

[80] Zińczuk J, Maciejczyk M, Zaręba K, et al (2019) Antioxidant Barrier, Redox Status, and Oxidative Damage to Biomolecules in Patients with Colorectal Cancer. Can Malondialdehyde and Catalase Be Markers of Colorectal Cancer Advancement? Biomolecules. 10.3390/biom9100637 10.3390/biom9100637 PMC6843197 31652642

[81] Shrivastava A, Mishra SP, Pradhan S (2021). An assessment of serum oxidative stress and antioxidant parameters in patients undergoing treatment for cervical cancer. Free Radic Biol Med.

[82] Li F, Liu W, Huo F (2021). Effect of self-controlled exercise on antioxidant activity of red blood cells and functional recovery of limbs in patients with breast cancer after rehabilitation. Iran J Public Health.

[83] Cousin S, Hollebecque A, Koscielny S (2014). Low skeletal muscle is associated with toxicity in patients included in phase I trials. Investig New Drugs.

[84] Cespedes Feliciano EM, Popuri K, Cobzas D (2020). Evaluation of automated computed tomography segmentation to assess body composition and mortality associations in cancer patients. J Cachexia Sarcopenia Muscle.

[85] Prado CM, Purcell SA, Laviano A (2020). Nutrition interventions to treat low muscle mass in cancer. J Cachexia Sarcopenia Muscle.

