# Supplementary material for: Sarcopenia and serum biomarkers of oxidative stress after a 6-month physical activity intervention in women with metastatic breast cancer: results from the ABLE feasibility trial
Source: Breast Cancer Res Treat. 2021 May 19;188(3):601–13. doi: 10.1007/s10549-021-06238-z (PMC8272711; doi:10.1007/s10549-021-06238-z)
Supplement: Supplementary file 1 — Supplementary file1 (DOCX 17 kb) [file 10549_2021_6238_MOESM1_ESM.docx]

**Supplementary data**

**Table 4**: Associations between sarcopenia at baseline, individual characteristics, physical condition and oxidative stress enzymes at baseline from the ABLE study (n=49).

| **Sarcopenic status** |  |  |  |  |
| --- | --- | --- | --- | --- |
| **Baseline** | All patients (n=47)  Mean (SD) or n (%) | Non-sarcopenic (n=22)  Mean (SD) or n (%) | Sarcopenic (n=25)  Mean (SD) or n (%) | p |
| Age (year), mean (SD) | 55.00 (10.18) | 53.43 (10.42) | 56.38 (9.97) | 0.27 |
| **ANTHROPOMETRICS** |  |  |  |  |
| Height (m), mean (SD) | 162.74 (6.19) | 161.41 (6.96) | 163.92 (5.30) | 0.26 |
| Weight (kg), mean (SD) | 69.36 (15.96) | 75.14 (17.32) | 64.28 (12.97) | **0.02** |
| BMI (kg/m²), mean (SD) | 26.17 (5.87) | 28.80 (6.39) | 23.86 (4.29) | **<0.01** |
| Underweight (<18.5 kg/m²), n (%) | 3.0 (6.4) | 1.0 (4.5) | 2.0 (8.0) | **0.04** |
| Normal weight (<25 kg/m²), n (%) | 19.0 (40.4) | 5.0 (22.7) | 14.0 (56.0) |  |
| Overweight (25–30 kg/m²), n (%) | 15.0 (31.9) | 8.0 (36.4) | 7.0 (28.0) |  |
| Obese (>30 kg/m²), n (%) | 10.0 (21.3) | 8.0 (36.4) | 2.0 (8.0) |  |
| **MUSCLE CHARACTERISTICS** |  |  |  |  |
| Cross sectional area at L3 (cm²), mean (SD) | 110.33 (16.42) | 122.89 (13.75) | 99.28 (8.92) | **<0.01** |
| SMG (arbitrary units), mean (SD) | 1389.95 (458.00) | 1567.09 (511.05) | 1567.09 (511.05) | **0.02** |
| Low SMD (<37.8HU) | 35.0 (74.5) | 16.0 (72.7) | 19.0 (76.0) | 0.80 |
| SMI (cm²/m²), mean (SD) | 41.72 (6.28) | 47.16 (4.48) | 36.94 (2.68) | **<0.01** |
| LBM (kg), mean (SD) | 39.16 (4.93) | 42.93 (4.13) | 35.84 (2.68) | **<0.01** |
| SMD (HU), mean (SD) | 33.16 (8.89) | 33.00 (9.42) | 33.31 (8.60) | 0.98 |
| **CLINICAL CARACTERISTICS** |  |  |  |  |
| Number of metastatic localizations, n (%) | 4.84 (3.00) | 4.13 (2.64) | 5.36 (3.26) | 0.51 |
| *De novo* metastatic breast cancer, n (%) | 14.0 (29.8) | 10.0 (45.5) | 4.0 (16.0) | **0.03** |
| Hormone therapy, n (%) | 28.0 (59.6) | 11 (50.0) | 17 (68.0) | 0.21 |
| Chemotherapy, n (%) | 25.0 (53.2) | 14.0 (63.6) | 11 (44.0) | 0.18 |
| **PHYSICAL FITNESS** |  |  |  |  |
| 6 minutes walking distance (m), mean (SD) | 445.03 (94.92) | 417.38 (103.37) | 469.23 (81.46) | 0.12 |
| Handgrip strength right (kg), mean (SD) | 26.18 (6.27) | 25.83 (6.38) | 26.48 (6.28) | 0.63 |
| Handgrip strength left (kg), mean (SD) | 30.34 (36.10) | 25.89 (5.89) | 33.90 (48.29) | 0.39 |
| Isometric quadriceps strength (N), mean (SD) | 194.84 (69.47) | 226.95 (78.87) | 169.71 (48.03) | **0.01** |
| **PHYSICAL ACTIVITY LEVEL** |  |  |  |  |
| Total physical activity (MET-minutes/week) | 2096.45 (2236.57) | 1854.14 (1928.22) | 2309.68 (2496.63) | 0.28 |
| Sitting time (min/week) | 2294.35 (1148.46) | 2368.64 (1197.23) | 2226.25 (1123.25) | 0.79 |
| Mean steps per day over a month, mean (SD) | 5226.98 (2775.33) | 4009.85 (2524.37) | 6038.41 (2709.99) | **0.05** |
| **ANTIOXIDANT ENZYMES** |  |  |  |  |
| CAT (µmol.min^-1^.L^-1^), mean (SD) | 34.21 (18.81) | 30.01 (8.66) | 37.75 (23.92) | 0.64 |
| GPx (mol.min^-1^.L^-1^), mean (SD) | 71.10 (22.31) | 66.51 (21.30) | 75.14 (22.82) | 0.17 |
| SOD (mol.min^-1^.L^-1^), mean (SD) | 9.27 (4.36) | 10.40 (5.01) | 8.31 (3.56) | 0.20 |
| **PROOXIDANT ENZYMES** |  |  |  |  |
| NADPH oxidase Leucocytes, mean (SD) | 0.22 (0.06) | 0.24 (0.06) | 0.21 (0.06) | 0.19 |
| MPO Plasma, mean (SD) | 111.51 (55.57) | 115.12 (62.59) | 108.47 (50.04) | 0.90 |
| MPO Leuco, mean (SD) | 15.89 (4.46) | 16.15 (4.47) | 15.67 (4.52) | 0.85 |
| **END PRODUCTS OF OS DAMAGES** |  |  |  |  |
| **LIPIDS** |  |  |  |  |
| MDA (µmol.L^-1^), mean (SD) | 11.43 (3.24) | 10.80 (3.21) | 11.96 (3.23) | 0.16 |
| **DNA** |  |  |  |  |
| 8-OHdG (µg.L-1), mean (SD) | 17.62 (18.40) | 17.76 (21.21) | 17.50 (16.13) | 0.40 |
| **PROTEIN** |  |  |  |  |
| AOPP (µmol.L^-1^), mean (SD) | 80.22 (56.06) | 68.91 (31.33) | 89.72 (69.78) | 0.99 |
| **INFLAMMATION** |  |  |  |  |
| IL6 (µmol.L^-1^), mean (SD) | 34.45 (9.23) | 35.66 (13.18) | 33.43 (3.59) | 0.72 |

Abbreviation: SMD, skeletal muscle density; SMI, skeletal muscle index; LBM, lean body mass; SMG, skeletal muscle gauge; BMI, body mass index CAT, catalase; GPx, gluatathione peroxidate; SOD, superoxide dismutase; MPO, myeloperoxidase; MDA, malondialdehyde, 8-OHdG, 8-hydroxydésoxyguanosine; AOPP, advanced oxidative protein products; IL, interleukin
